# A streamlined and predominantly diploid genome in the tiny marine green alga *Chloropicon primus*

**DOI:** 10.1038/s41467-019-12014-x

**Published:** 2019-09-06

**Authors:** Claude Lemieux, Monique Turmel, Christian Otis, Jean-François Pombert

**Affiliations:** 1 Département de biochimie, de microbiologie et de bio-informatique, Institut de Biologie Intégrative et des Systèmes, Université Laval, Québec, QC G1V 0A6 Canada; 2 Department of Biology, Illinois Institute of Technology, Chicago, IL 60616 USA

**Keywords:** Evolutionary biology, Comparative genomics, Genome evolution

## Abstract

Tiny marine green algae issued from two deep branches of the Chlorophyta, the Mamiellophyceae and Chloropicophyceae, dominate different regions of the oceans and play key roles in planktonic communities. Considering that the Mamiellophyceae is the sole lineage of prasinophyte algae that has been intensively investigated, the extent to which these two algal groups differ in their metabolic capacities and cellular processes is currently unknown. To address this gap of knowledge, we investigate here the nuclear genome sequence of a member of the Chloropicophyceae, *Chloropicon primus*. Among the main biological insights that emerge from this 17.4 Mb genome, we find an unexpected diploid structure for most chromosomes and a propionate detoxification pathway in green algae. Our results support the notion that separate events of genome minimization, which entailed differential losses of genes/pathways, have occurred in the Chloropicophyceae and Mamiellophyceae, suggesting different strategies of adaptation to oceanic environments.

## Abstract (web summary)

The Chloropicophyceae represent an important group of green algae in tropical oceans, but there is only limited genomic resource available. Here, the authors present the genome sequence of *Chloropicon primus*, revealing a diploid structure and the presence of a propionate detoxification pathway.

## Introduction

Green algae are morphologically, ecologically, and phylogenetically diverse, and represent one of the most successful groups of photosynthetic eukaryotes^1^; yet our knowledge about the genetic factors determining their ecological success is still limited. Soon after their emergence about 1000 Mya, early green algae split into two phyla: Chlorophyta, comprising the vast majority of extant species, and Streptophyta, comprising charophyte algae and all land plants^1–3^. In recent years, the genomes of a number of photosynthetic green algae, mainly from the Chlorophyta, have been sequenced to unravel the evolutionary trajectories followed by ancestral green algae^4–7^, decipher cellular mechanisms such as the basis of multicellularity^8–11^, pinpoint genes linked to adaptations to ecological niches^12–15^, and investigate metabolic networks, including routes for the biosynthesis of highly valuable compounds for the industry^16–19^.

**Fig. 1:**
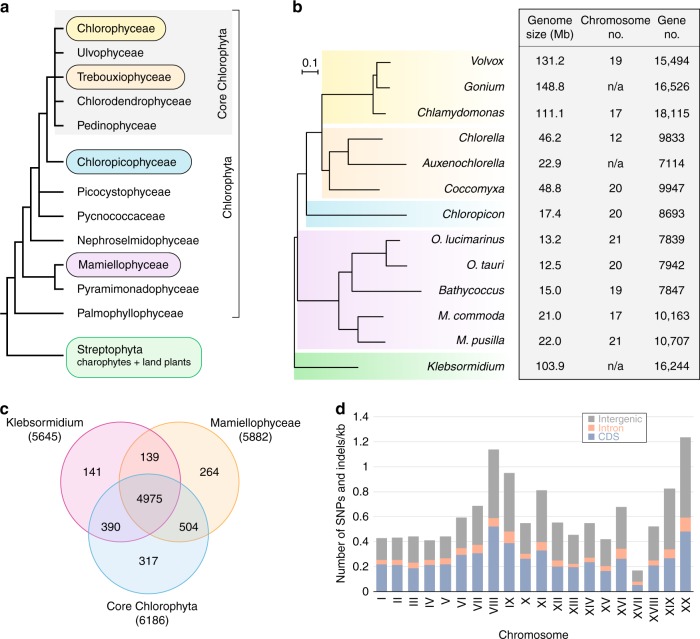
Phylogenetic position and genome features of *Chloropicon* relative to the 12 green algae selected for comparative analyses. **a** Schematic diagram depicting the main lineages of the Chloroplastida. The algae of our study group belong to the highlighted lineages. **b** Nuclear phylogenomic tree inferred from 792 concatenated proteins encoded by single-copy genes and comparison of genome sizes, numbers of chromosomes/DNA assemblies, and sizes of protein-coding gene repertoires. **c** Venn diagram showing the numbers of protein-coding genes that *Chloropicon* shares with the Mamiellophyceae, core chlorophytes and *Klebsormidium*. **d** Density of SNPs and small indels in coding (blue bars) and non-coding (intron, orange bars; intergenic sequences, gray bars) regions of individual *Chloropicon* chromosomes. The source data of **d** are provided as a Source Data file

Known for their important role in the global carbon cycle, the green algae traditionally considered as prasinophytes constitute a paraphyletic assemblage of unicellular, predominantly marine organisms at the base of the Chlorophyta^2,20,21^ (Fig. 1a). Decoding their genomes is thus expected to yield important insights into the nature of the first green algae and their patterns of diversification. It has been suggested that the earliest-diverging green algae were scaly flagellates that were able to capture bacteria, although only a few extant prasinophytes such as *Cymbomonas* (Pyramimonadophyceae) exhibit a phagotrophic lifestyle^1^. Among the independent prasinophyte lineages that have been identified so far, at least five (Prasinococcales, Mamiellophyceae, Pycnococcaceae, Picocystophyceae, and Chloropicophyceae) comprise coccoid (no flagella, no scales) species of small size (≤5 μm in diameter). A highly reduced, coccoid growth form is thought to confer a distinct advantage to planktonic algae because the resulting higher surface area to volume ratio enhances the efficiency of nutrient uptake, thus increasing competitiveness in an environment poor in nutrients; furthermore, a reduced size helps to escape predators and promotes buoyancy^21–23^.

The Mamiellophyceae comprises the smallest known free-living eukaryote, the coccoid *Ostreococcus tauri*, and is the only prasinophyte lineage for which complete nuclear genomes are currently available (Fig. 1b). The tiny algae belonging to the three genera from the order Mamiellales—*Ostreococcus*, *Micromonas* (no scales and one flagellum), and *Bathycoccus* (scales but no flagella)—are typically found in coastal waters although they have been observed in open oceanic waters^21^. For each of these genera, cryptic species derived from separate clades or strains representing different ecotypes were targeted for genome analysis^4–7,24,25^. All examined mamiellophyceans possess reduced genomes (12–22 Mb with only ~8000–11,000 predicted genes, Fig. 1b) compared to freshwater/terrestrial green algae and the prasinophyte *Cymbomonas tetramitiformis*^26^, implying that genome minimization occurred along cell reduction and simplification in the course of prasinophyte evolution^22^. The *Ostreococcus* genus exhibits the smallest genomes among sequenced green algae and as in other unicellular green algae, each chromosome is present in one copy. Within each genus of the Mamiellophyceae, species divergence or adaptation to ecological niches was linked to dynamic genome evolution, with dispensable and fast-evolving genes predominantly located in the smallest of the two outlier chromosomes displaying lower GC content. The small outlier chromosome is thought to be specialized in defense against viruses^27^, while the larger appears to contain the sex-determining region (SDR)^4,6,28^.

Recently, a late-diverging lineage of pico/nano-prasinophytes, named Chloropicophyceae, has emerged as playing a key role in marine phytoplankton communities, especially in moderately oligotrophic waters^29,30^. This genetically diversified lineage, which includes two genera (*Chloropicon* and *Chloroparvula*), occupies a sister position relative to the core Chlorophyta^2,20,30,31^ (Fig. 1a) and although its members represent the dominant green algal group in open oceanic waters of tropical regions, little is known about their life cycle (sexual reproduction has not been observed) and their biochemical and physiological features.

In the present study, we report the nuclear genome sequence of *Chloropicon primus*, a member of the Chloropicophyceae. Our analyses of this 17 Mb genome provide important insights into the biology of this picoalga and the extent to which the Chloropicophyceae and Mamiellophyceae differ in their metabolic capacities and cellular processes. Major features distinguishing *Chloropicon* from most other sequenced green algae include a predominantly diploid structure, with one of the 20 chromosomes present in three copies, and a metabolic pathway for degradation of propionate.

## Results

### The *Chloropicon* nuclear genome is small and compact

**Fig. 2:**
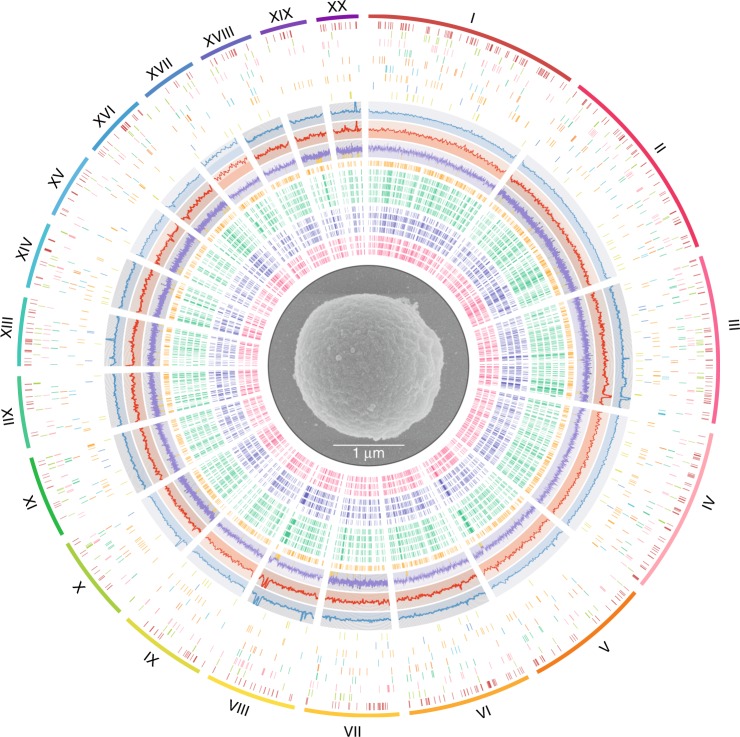
Physical map of the *Chloropicon* genome. Center. Scanning transmission electron microscopic image of this alga (provided by W. Eikrem and T. Pollina, University of Oslo). Outer ring. The 20 chromosomes were labeled from I to XX (largest to smallest; magenta to purple). Inner rings 2–10. Distribution of *Chloropicon* genes across the KEGG metabolic pathways. From outside to inside; (2) genetic information processing, (3) environmental information processing, (4) cellular processes, (5) carbohydrate metabolism, (6) energy metabolism, (7) lipid metabolism, (8) amino acid, and nucleotide metabolism, (9) glycan biosynthesis and metabolism, (10) cofactors, vitamins, terpenoids, and polyketides metabolism. Inner ring 11. Illumina (paired-end + mate pair) sequencing depth below/above average (370×) is colored in blue/light blue (*y*-axis min-max: 100–700). Inner ring 12. PacBio sequencing depth below/above average (73×) is colored in red/light red (*y*-axis min-max: 0–150). Inner ring 13. GC content of *Chloropicon* chromosomes. Background is colored in purple/light purple for values below/above average (57.5 %) (*y*-axis min-max: 37.5–77.5). Regions displaying GC content lower than 48% or higher than 70% are highlighted underneath by gold and gray lines, respectively. Inner rings 14–26. Genes shared between *Chloropicon* and other green algal genomes; (14) *K. flaccidum* (gold), (15–19) *O. tauri*, *O. lucimarinus*, *M. pusilla*, *M. commoda* and *B. prasinos* (green), [20–23] *Helicosporidium* sp., *C. subellipsoidea*, *C. variabilis* and *A. protothecoides* (blue), [24–26] *G. pectorale*, *C. reinhardtii* and *V. carteri* (magenta)

The *Chloropicon* nuclear genome was assembled into 20 chromosomes, all of which were sequenced from telomere to telomere, with CCTAAAAA as the canonical sequence of the telomeric unit. Sizes of the individual chromosomes vary from 0.37 to 1.88 Mb, for a total genome size of 17,400,691 bp, an intermediate value between the *Bathycoccus* and *Micromonas* genomes (Fig. 1b). The *Chloropicon* genome is dense in coding sequences, with averages of 0.50 gene per kb and 0.50 predicted intron (average size of 152 bp) per gene (Supplementary Data 1), and the repeats identified in non-coding regions represent only 4% of the genome. No major difference in overall GC content was noted across chromosomes; however, short regions of a few chromosomes—in particular, the regions containing polyketide synthase genes in chromosomes 3, 5, and 8—are characterized by a lower GC content (Fig. 2).

A set of 8639 protein-coding genes were predicted in the *Chloropicon* genome based on the curated sum of evidence built from gene prediction, mapping of cDNAs from normalized libraries, and homology searches (Supplementary Data 2). Of these genes, 7294 (84.4%) were found to be expressed at an average RNAseq depth ≥1.0 (Supplementary Data 2) and 4765 were assigned putative functions (cutoff *E*-value = 1.0E-10), with 3723 further assigned to KEGG orthologs. Nearly 78% of the predicted *Chloropicon* proteins are shared with the 12 other green algae used in our genome comparisons (Fig. 1c). Of the 1909 proteins unique to *Chloropicon*, only 163 featured putative functions as inferred using our annotation pipeline and GhostKOALA, and 82, 3, and 145 yielded strongest hits to proteins of bacteria, archaea, and eukaryotes from non-green lineages, respectively, in BLASTP searches (cutoff *E*-value = 1.0E-10) against the nr database of the National Center for Biotechnology Information (NCBI).

As an independent method to assess the quality and completeness of our genome assembly and annotation, we queried using BUSCO^32^ the annotated proteins of *Chloropicon* and 13 other green algae against the OrthoDB v10 database, which contains the collection of 2168 near-universal single-copy orthologs found in the Chlorophyta (Supplementary Fig. 1). The *Chloropicon* proteins accounted for 82.5% of this collection, a value slightly lower than those obtained for *Ostreococcus tauri* (88.9%), *Bathycoccus* (86.9%), and *Gonium* (86.9%).

As observed for the Mamiellales, there is a limited number of gene families in *Chloropicon*, with copies of identical or very similar sequences often occurring as tandem repeats. The largest families include genes that are dispersed throughout the genome and these encode polyketide synthases (12 members, Supplementary Data 3), sialyltransferases (59 members, Supplementary Data 4), guanylyl cyclases (30 members, Supplementary Data 5), and proteins similar to transposases of the IS605 insertion sequence family (25 members, Supplementary Data 6).

In terms of gene synteny, the *Chloropicon* genome is highly divergent compared to previously sequenced green algal genomes (Supplementary Fig. 2). The largest syntenic block, a suite of only 27 gene pairs, was found to be shared with the trebouxiophyte *Coccomyxa*. This observation contrasts with the high degree of gene colocalization within the chromosomes of mamiellaleans (Supplementary Fig. 2).

### Most *Chloropicon* chromosomes have a diploid structure

**Fig. 3:**
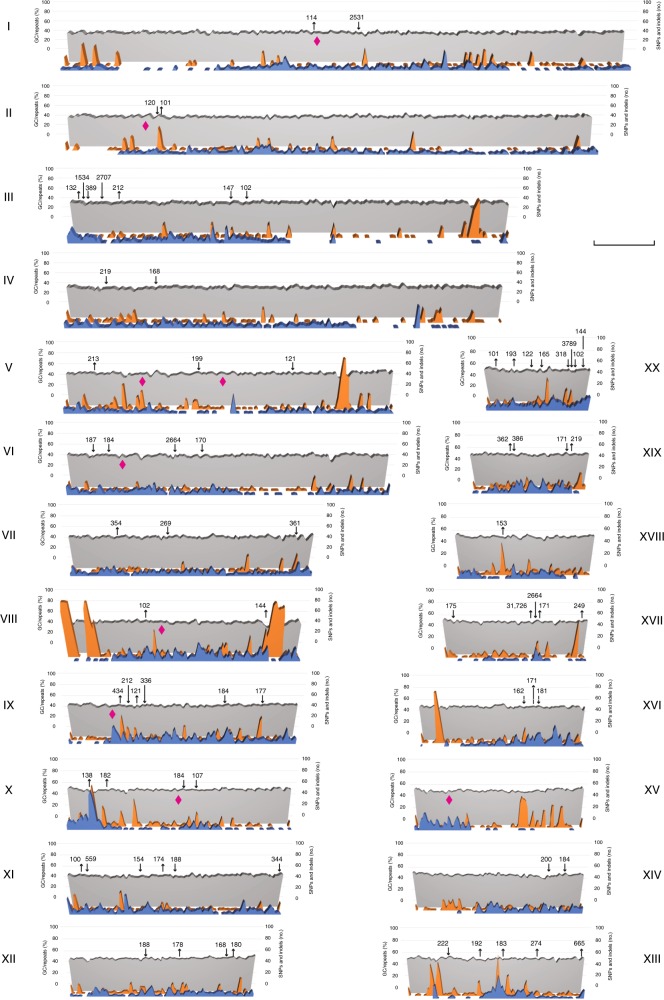
Variation in GC content and distribution of polymorphic loci and repeats throughout individual *Chloropicon* chromosomes. The curves shown in blue were obtained by plotting the numbers of SNPs and small indels present in sliding windows of 10,000 nt with a 5000-nt step; dispersed repeats were masked before the identification of these polymorphisms. The orange curves depict the percentages of nucleotides that are part of repeats in the same windows, while the gray curves depict the percentages of nucleotides with guanine and cytosine bases. The locations of indels ≥100 bp are denoted by arrows pointing up (deletions) and down (insertions), and the loci of putative *MID* genes by magenta diamonds. Chromosomes are labeled with Roman numbers and their lengths are represented to scale on the corresponding *x*-axis. The scale bar represents 200 kb

Polymorphisms are pervasive in the *Chloropicon* genome, an unexpected discovery considering that all unicellular green algae analyzed so far are haploids and that the *Chloropicon* culture was derived from a clone. A total of 7282 single-nucleotide polymorphisms (SNPs) and 2048 small insertions/deletions (indels) were identified by aligning the Illumina reads against the genome sequence, for an average of 0.54 variants per kb (i.e. 0.05% divergence), with minimum and maximum values of 0.17 and 1.24 for chromosomes XVII and XX, respectively (Fig. 1d and Supplementary Data 7). Each polymorphic site features two alleles and two allelic combinations are readily distinguishable in aligned reads spanning two or more SNP sites. Except for a few regions, the polymorphic sites are distributed uniformly along each chromosome (Fig. 3). In coding regions which represent 73.8% of the genome, SNPs and small indels showed a 3.5- to 3.8-fold lower density than in intergenic regions and introns, respectively (Fig. 1d). Furthermore, 73 indels larger than 100 bp were identified by mapping PacBio reads onto the chromosomal assemblies (Fig. 3 and Supplementary Data 8).

**Fig. 4:**
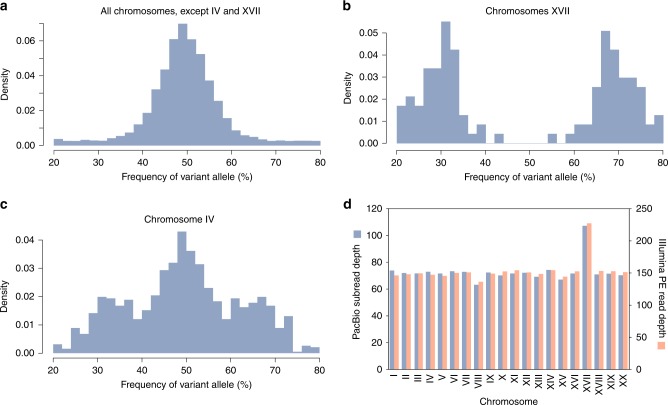
Allelic frequencies of SNPs and small indels, and coverage depth of sequencing reads on *Chloropicon* chromosomes. **a** Allelic frequency distribution observed for all chromosomes, except IV and XVII. **b** Allelic frequency distribution observed for chromosome XVII. **c** Allelic frequency distribution observed for chromosome IV. **d** Mean PacBio and Illumina (paired-end) sequencing depths of each chromosome. Source data are provided as a Source Data file

**Fig. 5:**
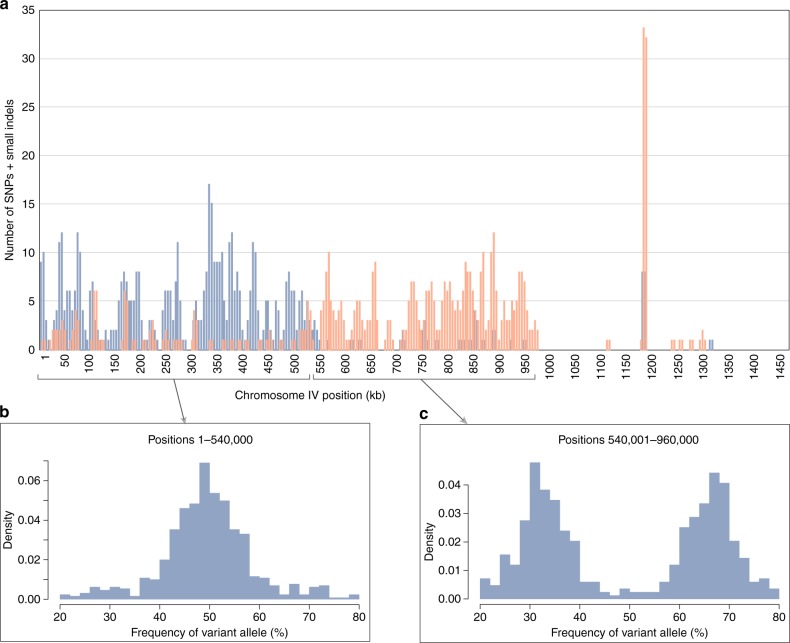
Clustering of SNPs and small indels strongly departing from the 1:1 allelic ratio on chromosome IV. **a** Distribution of polymorphic sites with allelic frequencies falling in the following categories: 40–60% (blue) and 20–39% + 61–80% (orange). **b** Allelic frequency distribution observed in the segment delimited by positions 1–540,000. **c** Allelic frequency distribution observed in the segment delimited by positions 540,001–960,000. Source data are provided as a Source Data file

For all chromosomes, except IV and XVII, plots of the allelic frequencies observed at each polymorphic locus displayed a bell shape distribution with a mean of 50% (i.e. 1:1 allelic ratio), a result consistent with a diploid structure (Fig. 4a). However, the distribution observed for chromosome XVII was bimodal with means of 33 and 66%, as predicted for a trisomic state (Fig. 4b). The latter interpretation is supported by the finding that the coverage depth estimated for chromosome XVII was 1.5-fold greater relative to other chromosomes (Fig. 4d). With regards to chromosome IV, the allelic frequency distribution appeared to be a composite of the two patterns just described (Fig. 4c), and strikingly, the polymorphic loci strongly departing from the 1:1 ratio are tightly clustered in a region covering 420 kb (Fig. 5). Note that shorter segments on other chromosomes also revealed aberrant allelic ratios (Supplementary Fig. 3).

### Cellular copy number of *Chloropicon* organelle genomes

No polymorphisms were detected by mapping the sequencing reads generated in the course of this study against the 64.3-kb plastome and 37.9-kb mitogenome reference sequences available for *C*. *primus*^20,31^. Comparison of the coverage depth of PacBio subreads (449x and 253x for the plastome and mitogenome, respectively) to the mean PacBio sequencing depth obtained for the 19 chromosomes present in two copies (73×) enabled us to estimate that each *Chloropicon* cell, which contains a single chloroplast and a single mitochondrion, houses about 12 plastid and seven mitochondrial DNA molecules. These values are respectively 7- and 9-fold lower compared to those reported for *C*. *reinhardtii* (over 80 copies of the 205-kb plastome and about 130 copies of the 15.8-kb mitogenome as estimated by Gallaher et al.^33^); however, they are higher compared to the estimates provided for the Mamiellophyceae (4–6 copies for cell)^22^.

### Genes involved in meiosis and production of flagella

As reported for most of the chlorophytes previously investigated, the meiosis-specific genes encoding SPO11–2, DCM1, Rad51c and XRCC3, MSH4, and MSH5 are present in *Chloropicon* and there is no indication that these genes are pseudogenes. We detected nil or very low levels of expression of these genes under vegetative growth (Supplementary Data 9).

Although vegetative cells of *Chloropicon* lack flagella, they possess an almost full complement of protein genes known to be involved in flagellar structure and maintenance (Supplementary Data 10). Other non-motile chlorophytes such as *Ostreococcus*, *Bathycoccus*, and *Chlorella* have also retained protein genes of this nature, but unlike *Chloropicon*, they are missing numerous genes involved in intraflagellar transport and radial-spoke formation. Furthermore, although no scales were distinguished on the three-layer wall of *Chloropicon*^30^, our analyses unveiled a large number of N- and O-glycan modifying enzymes that have been implicated in scale formation based on their specific occurrence and/or enrichment in the scale-bearing prasinophytes *Bathycoccus* and *Dolichomastix*^24^. Notably, 59 genes encoding sialyltransferases and nine encoding sialidases were identified, the great majority of which are silent or transcribed at very low levels under the growth conditions used (Supplementary Data 4).

### Search for a candidate chromosome carrying the SDR

The primarily diploid and heterozygous structure of the *Chloropicon* genome suggests that fusion between haploid gametes of different genotypes yielded a diploid individual that reproduced subsequently by mitosis. If gametes of distinct mating types (*mt*^*+*^ and *mt*^*−*^) were engaged in this mating as is the case for heterothallic volvocine algae, one would expect the *Chloropicon* SDR (mating-type locus) to be heterozygous in the same way as vegetative diploids of *Chlamydomonas* and to exhibit a significant difference in size and gene content between the two homologous SDR copies; moreover, the copy derived from the *mt*^−^ gamete would be expected to carry the master sex determination *MID* gene coding for a transcription factor with a DNA-binding RWP-RK domain^34^. As the cause or consequence of recombination suppression, the SDR region usually exhibits genomic rearrangements and a high density of repeated sequences. Our genome analyses revealed nine *Chloropicon* genes encoding putative RWP-RK transcription factors, but none was part of a large indel region or a region exceptionally rich in repeats or polymorphisms (Fig. 3), and sequence alignment of motifs 1 and 2 of these proteins^35^ with those present in the Mid proteins of volvocine algae revealed no obvious ortholog of Mid (Supplementary Fig. 4).

### Genes for light-harvesting complex proteins

The chlorophyll- and carotenoid-binding proteins making up the light-harvesting complexes associated with photosystem II (LHCII) and photosystem I underwent considerable changes during the evolution of the Chloroplastida^36,37^. The LHCII proteins (Lhcb) are especially diverse. Besides the invariably conserved minor Lhcb4 (CP29) and Lhcb5 (CP26) proteins, mamiellophyceans feature at least five prasinophyte-specific LHCII proteins (Lhcp)^37,38^, whose evolutionary origin predates the divergence of streptophytes and chlorophytes^37^. In contrast, all six *Chloropicon* Lhcb proteins are typical of core chlorophytes (Supplementary Data 11).

### Carotenoid biosynthesis genes

The Chloropicophyceae contain the basic set of pigments found in the Chlorophyceae (neoxanthin, violaxanthin, lutein, zeaxanthin, antheraxanthin, and β-β carotene) as well as loroxanthin^30,39^. In addition, like *Haematococcus pluvialis*^40^ and *Chromochloris zofingiensis*^41^, most members of the Chloropicophyceae, including *C. primus*, produce astaxanthin^30,39^. On the other hand, carotenoids characteristic of the prasino-3 group (prasinoxanthin, dihydrolutein, uriolide, micromonal, and micromonol) are found in the Mamiellophyceae^42^.

We identified the complete set of *Chloropicon* genes responsible for carotenoid biosynthesis (Supplementary Data 12 and Supplementary Fig. 5). As in land plant and core chlorophytes, two desaturases (Pds and Zds) and two *cis*-*trans* isomerases (Z-Iso and CrtIso) catalyze the four steps of dehydrogenation that are required for converting 15-*cis* phytoene into all-*trans* lycopene. In addition, there is a gene encoding a bacterial/fungal-type phytoene desaturase (CrtI), an enzyme performing multiple dehydrogenation steps. All members of the Mamiellophyceae are missing a number of genes in the pathway typically found in both chlorophytes and streptophytes.

The proteins known to participate directly in astaxanthin synthesis, namely beta-carotene ketolase (Bkt) and beta-carotene hydroxylase (Chyb), are encoded by single-copy genes in *Chloropicon*. The order in which they function in *H*. *pluvialis* and *C*. *zofingiensis* is still uncertain^40,41^. Considering that only *chyb* has been retained in the Mamiellophyceae, we speculate that its protein product is required to synthesize zeaxanthin, a precursor of both violaxanthin and neoxanthin; hence, assuming that the *Chloropicon* Chyb has the same substrate specificity, we propose that subsequent ketolation of zeaxanthin by Bkt yields astaxanthin (Supplementary Fig. 5).

### Thiamine biosynthesis genes

We inferred that *Chloropicon* has the ability to synthesize thiamine (vitamin B1) (Supplementary Data 13 and Supplementary Fig. 6). Considering that the availability of exogenous vitamins is limited in open oceans, the retention of this biosynthesis pathway may help explain its ubiquitous presence of *Chloropicon* across the globe. In the form of thiamine diphosphate (TPP), vitamin B1 is an essential cofactor for energy generation and general metabolism that is produced via the coupling of pyrimidine and thiazole precursors, namely hydroxymethylpyrimidine pyrophosphate (HMP-PP) and hydroxyethyl-thiazole phosphate (HET-P) or 5-(2-hydroxyethyl)-4-methyl-1,3-thiazole-2-carboxylic acid (cHET-P)^43,44^. Catalyzed by the bifunctional enzyme TH1, this reaction results in thiamine monophosphate, a precursor that is subsequently converted to TPP by the kinase TPK. The pyrimidine moiety is produced in reactions catalyzed by ThiC and TH1, whereas the thiazole group is synthesized by Thi4^43^.

In *Ostreococcus* and *Micromonas*, *thiC*, *thi4* and *th1* are pseudogenes^43^. To survive, these picoeukaryotes are dependent on exogenous vitamin B1 or related micronutrients supplied by B1-synthesizing marine bacteria or eukaryotic phytoplankton^44,45^. It has been recently shown that the thiazole precursor cHET is a key micronutrient in the Mamiellophyceae and that its utilization is dependent upon the activity of the kinase ThiM^44^. A *Chloropicon* homolog of this protein could not be detected by BlastP searches.

### Catabolism of branched-chain amino acids

In plants, leucine, isoleucine, and valine are known to play a crucial role as alternative sources of energy under carbohydrate starvation or prolonged darkness^46^ (see Supplementary Fig. 7 for the branched-chain amino acids, BCAA, degradation pathways). This auxiliary supply of energy is generated in the mitochondrion. The *Chloropicon* genome potentially encodes all the proteins necessary to degrade the three BCAAs and similarly, complete BCAA catabolic pathways were inferred for the trebouxiophytes *Helicosporidium, Auxenochlorella*, and *Coccomyxa* (Supplementary Fig. 7 and Supplementary Data 14). In the case of the four other examined core chlorophytes, only one enzyme (enoyl-CoA hydratase, K07511) in the valine and isoleucine catabolic pathways was not detected using GhostKOALA. In contrast, numerous proteins in the degradation pathways of the three BCAAs were not retrieved in the Mamiellophyceae.

### A metabolic pathway unique to *Chloropicon*

**Fig. 6:**
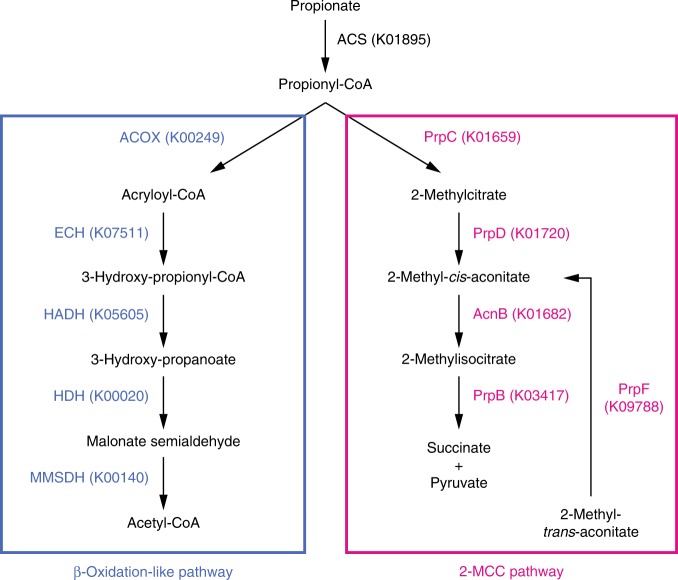
*Chloropicon* proteins predicted to participate in the degradation of propionate via the modified β-oxidation and 2-MCC pathways. Supplementary Data 15 report the loci of the encoding genes, the presence/absence of homologs in other green algae and additional information, including expression data and predicted subcellular localization. PrpC, 2-methylcitrate synthase [EC:2.3.3.5]; PrpD, 2-methylcitrate dehydratase [EC:4.2.1.79]; AcnB, aconitate hydratase 2/2-methylisocitrate dehydratase [EC:4.2.1.99]; PrpB, methylisocitrate lyase [EC:4.1.3.30]; PrpF, 2-methylaconitate *cis-trans*-isomerase [EC:5.3.3.-]; ACOX, acyl-CoA dehydrogenase [EC:1.3.8.7]; ECH, enoyl-CoA hydratase [EC:4.2.1.17]; HADH, 3-hydroxyisobutyryl-CoA hydrolase [EC:3.1.2.4]; HDH, 3-hydroxyisobutyrate dehydrogenase/3-hydroxypropionate dehydrogenase [EC:1.1.1.31]; MMSDH, malonate-semialdehyde dehydrogenase (acetylating)/methylmalonate-semialdehyde dehydrogenase [EC:1.2.1.18 1.2.1.27]; ACS, acetyl-CoA synthetase [EC:6.2.1.1]

Among the proteins unique to *Chloropicon*, we uncovered the complete set of enzymes performing the degradation of propionate via the 2-methylcitrate cycle (2-MCC)^47^, namely ACS, PrpC, PrpD, AcnB, and PrpB (Fig. 6 and Supplementary Data 15). The gene encoding PrpF was also identified, albeit this enzyme is not likely to function in an alternative branch of the 2-MCC due to the absence of the upstream gene product (AcnD). To ensure that the 2-MCC was not previously overlooked in the Chloroplastida, we examined all green algal genomes sequenced to date using GhostKOALA and did not identify PrpC, PrpB, and PrpF. Although PrpD was retrieved in the *Ostreococcus* and *Micromonas* genera, the latter protein sequences were highly divergent from that of *Chloropicon* (*E*-value ≥ 1.0E-10), an unanticipated result considering the very strong level of similarity (*E*-value = 0) observed for the *Chloropicon* and mamiellophycean orthologs of the bifunctional AcnB enzyme (aconitate hydratase in the TCA cycle and 2-methylisocitrate dehydratase in the 2-MCC). Congruent with BlastP searches, phylogenetic trees inferred from the *Chloropicon* PrpB, PrpC, PrpD, and PrpF proteins identified different bacteria and/or non-photosynthetic eukaryotes as their closest relatives (Supplementary Fig. 8), highlighting complex evolutionary histories for the genes encoding these proteins which likely involved horizontal transfer events from bacteria and multiple losses in eukaryotic lineages. Reminiscent of the gene organization found in bacteria^48^, *prpB*, *prpD,* and *prpC* are closely linked on chromosome V of the *Chloropicon* genome (Supplementary Data 2).

## Discussion

Like mamiellalean genomes, the *Chloropicon* genome has a small size and is densely packed with coding sequences, with a minimum of gene redundancy; however, extensive differences are seen at the gene content and gene arrangement levels. *Chloropicon* exhibits genes/pathways that are present in core chlorophytes but not in mamiellaleans, and conversely, mamiellaleans exhibits genes/pathways that are not present in *Chloropicon*, suggesting that the two groups of picoalgae adopted different strategies to adapt to their oceanic environments. Key genes belonging to the former category include those encoding pigment-binding proteins of light-harvesting complexes, proteins required for synthesis of carotenoid and thiamine, and enzymes participating in degradation of BCAAs.

The common ancestor of the Chloropicophyceae and Mamiellophyceae emerged about 950 Mya^49^ and like the algae belonging to the sister class of the Mamiellophyceae (Pyramimonadophyceae), was presumably a scaly flagellate with a large cell body and a complex internal morphology that enabled a phago-mixotrophic mode of nutrition^50^. A draft genome of the phagophotrophic *Cymbomonas tetramitiformis* highlighted a considerably greater genome size (850–1200 Mb) and gene repertoire (37,366 gene models) compared to those of *Chloropicon* and the Mamiellales^50^. Our study thus reinforces the view that several independent events of genome minimizations, entailing differential gene losses, occurred during prasinophyte diversification.

Analyses of allelic ratios at polymorphic sites and of chromosome coverage depths revealed that all 20 *Chloropicon* chromosomes, except the trisomic chromosome XVII, are present in two copies. Further supporting this conclusion is the finding that the DNA content previously estimated for *Chloropicon* cells by flow cytometry predicted a genome size of about 45 Mb, which is twice larger than our genome assembly^30^. While diploid strains of *C. reinhardtii* that reproduce asexually can be generated in the laboratory, a predominantly diploid phase of growth (i.e. diplontic life cycle) has not been observed among natural isolates of unicellular green algae^51^, but it is prevalent in other groups of unicellular algae such as the diatoms^52^. In green algae, diploid vegetative cells have been documented solely for ulvophytes with a haplo-diplontic life cycle^52^.

Despite its higher metabolic cost, diploidy (i.e. a life cycle with a predominantly diploid growth phase) has been traditionally viewed as evolutionary favored over haploidy. By allowing the masking of nearly all deleterious mutations arising in the genome, diploidy has been proposed to confer higher organismal fitness and a larger pool of mutant alleles that may help adaptation to new environments^52,53^. However, given that more mutations arise in diploids than haploids and that selection is less efficient, theoretical studies have shown that a dominant diploid phase is disadvantageous under certain circumstances, e.g. when the environment is static, and is tolerated as long as deleterious mutations are masked and as long as there is enough sex and recombination in the population. By contrast, newly arising deleterious mutations are more efficiently eliminated in haploids and beneficial mutations spread more easily, increasing fitness at equilibrium. Furthermore, haploid cells may have a nutritional advantage over diploid cells because they are often smaller than diploids and thus have a higher surface area to volume ratio; in the yeast *Saccharomyces cerevisiae*, it has been experimentally shown that haploids are more suitable than diploids for some conditions of nutrient limitations^52,53^. In this context, it is noteworthy that different isolates of *Chloropicon roscoffensis* were shown to differ in DNA content and that two strains from the low genome size (20 Mb) group also display a 25% smaller average cell diameter compared to strains from the high genome size (42 Mb) group^30^.

Only a few green algae, including the prasinophyte *Nephroselmis olivacea*^54^, have been observed to engage in sex in nature or laboratory conditions. In line with the idea that sexual reproduction is nearly universal in eukaryotes, genomics and/or population genetics studies algae sequenced have revealed indirect evidence for the capacity of sexual reproduction in most of the green so far^4,6,13,19,28,55^, including *Chloropicon*. The conservation of genes related to meiosis/sex and flagellar structure and maintenance (flagella play a role in the recognition of mating partners) suggests that *Chloropicon* had a past history of sex or has retained the ability to produce haploid gametes whose fusion gives rise to new diploid genotypes with purged deleterious alleles. Our data on the expression of sialyltransferase genes also raise the possibility that there exists a life-cycle phase in which cells exhibit sialylated scales or scale-like structures. This phase might be linked to sexual reproduction, given that a sialyltransferase activity believed to be important for cell-cell interaction has been identified on the external surface of gametes in *Chlamydomonas moewusii*^56^.

Assuming that *Chloropicon* is heterothallic (mating occurring between gametes with genetically determined mating-types), one would expect that the SDR would be diploid in the same way as vegetative diploids of *Chlamydomonas* (with one copy of each *mt*^*+*^ and *mt*^*−*^ loci) and that, as documented for heterothallic volvocine algae^34^, the dominant sex-determining gene *MID* gene would be located within a genomic region enriched in indels and SNPs. Our failure to identify a region with these characteristics may be interpreted as indicating that the *Chloropicon mt*^*+*^ and *mt*^*−*^ loci are short and highly similar to one another. However, such an exceptionally high level of similarity seems unlikely considering our current knowledge about SDR architecture^34^ and for this reason, the hypothesis that *Chloropicon* is homothallic (self-fertile) cannot be ruled out. In volvocine lineages, homothallism repeatedly evolved from heterothallic ancestors^57^ and for a homothallic species of *Volvox*, it has been shown that epigenetic control of expression of the dominant *MID* is probably involved in the formation of male and female gametes within a single clonal culture, although no information is currently available on the genomic region corresponding to the *MT* locus of heterothallic relatives^58^.

Aneuploidy is a widespread phenomenon in unicellulars such as *S. cerevisiae* and the primarily asexual fungus *Candida albicans* (an heterozygous diploid like *C. primus*) that imposes a burden on the cell but provides a fitness advantage under certain growth or stressful conditions^59,60^. *Chloropicon* might have gained a third copy of chromosome XVII during the period of asexual growth following isolation of the original *Chloropicon* CCMP1205 strain in 1965. Additional *C*. *primus* isolates will need to be investigated to determine the time of emergence of this trisomy.

The significant deviations from the 1:1 allelic ratio observed on chromosome IV and other chromosomal regions might be the result of biased gene conversion events (LOH events) arising from mitotic recombination, followed by segregation of distinct genotypes during further growth of *Chloropicon* CCMP1205. In asexual diploid eukaryotes, LOH is thought to provide a mechanism for removing deleterious mutations and thus compensate for a lack of sex^60–63^.

Among the proteins unique to *Chloropicon*, we uncovered the complete set of enzymes performing the degradation of propionyl-CoA via the 2-MCC. Propionate, in the form of propionyl-CoA, is a toxic metabolite that is produced by several pathways: breakdown of several amino acids (methionine, threonine, and the BCAAs isoleucine and valine), β-oxidation of odd-chain fatty acids, and catabolism of branched fatty acids, such as phytanic acid which is derived from the degradation of chlorophyll. Propionyl-CoA has been shown to interfere with enzymes of the central metabolism^64^ and to inhibit polyketide synthesis^65^. In many prokaryotes and some eukaryotes, propionate also serves as a carbon source^48,64^. Organisms have evolved a variety of mechanisms to convert propionyl-CoA into products that can be further metabolized via the TCA cycle^47^: a propionate carboxylation pathway dependent on vitamin B12 that produces succinyl-CoA^66^, a modified β-oxidation pathway leading to acetyl-CoA and CO_2_, and the 2-MCC pathway converting propionate into succinate and pyruvate.

It remains unclear whether the 2-MCC is the only metabolic route available to *Chloropicon* for propionyl-CoA detoxification. We identified candidate genes that potentially encode all the proteins catalyzing the reactions of the modified β-oxidation pathway (Fig. 6 and Supplementary Data 15); however, all are also required for BCAA catabolism (Supplementary Data 14 and Supplementary Fig. 7) and contrary to the model plant *Arabidopsis*^67,68^, PredAlgo and TargetP analyses predicted that the putative enoyl-CoA hydratase (ECH, K07511) responsible for the production of the key metabolite β-hydroxypropionate is located in the mitochondrion instead of the peroxisome.

Considering the scarcity of nutrients available in open oceans compared to coastal waters, the ability to recycle propionate for other metabolic purposes such as a source of carbon and energy could confer a clear benefit to *Chloropicon*. To our knowledge, the colorless obligatory-heterotrophic trebouxiophyte *Prototheca zopfii* is the sole green alga that has been reported to utilize propionate as carbon source, and in this case, this organic acid appears to be metabolized by the modified β-oxidation pathway^69^. In contrast, no propionate catabolic pathway appears to be present in the Mamiellophyceae; given their inability to catabolize BCAAs using the pathways typically found in eukaryotes, these prasinophytes can perhaps dispense with propionyl-CoA detoxification.

As reported for plants, BCAA degradation might contribute to cell fitness in the Chloropicophyceae and core chlorophytes under carbohydrate starvation or prolonged darkness. The apparent absence of the typical BCAA catabolic pathways in the Mamiellophyceae is intriguing; perhaps these prasinophytes possess alternative pathways to degrade BCAAs that are not available to most eukaryotes, such as the Ehrlich pathway used by *S. cerevisiae* during fermentation^70^. Alternatively, mamiellophyceans might be less dependent than *Chloropicon* and core chlorophytes upon BCAA catabolism under nutrient- or light-limiting conditions if they are more efficient at producing reserves of energy in the forms of carbohydrates or lipids or at harvesting energy from alternative metabolites. In this context, it is worth reporting that our genome analyses predict that *Chloropicon* utilizes a more limited range of intermediates than mamiellophyceans during anaerobic fermentation metabolism^14^ (Supplementary Data 16) and that unlike mamiellophyceans^6^, it cannot use a C4-like mechanism to concentrate CO_2_ due to the absence of the gene encoding a pyruvate orthophosphate dikinase (EC 2.7.9.1).

The differences in Lhcb proteins between the Mamiellophyceae and Chloropicophyceae are possibly linked to differing pigment compositions. These algal classes exhibit distinct carotenoid profiles and unlike the Mamiellophyceae, the Chloropicophyceae lack the chlorophyll *c* derivative Mg-DVP^42^. The capacity to produce astaxanthin may help *Chloropicon* to thrive under high light intensity. This ketocarotenoid is thought to confer protection by absorbing excess light and quenching reactive oxygen species^17,40^.

Another key feature of the *Chloropicon* genome is the large number of guanylyl cyclase genes, which suggests an important role in cellular signaling and regulation of gene expression^71^. In *C*. *reinhardtii*, studies of two of the six soluble isoforms of guanylate cyclase present in this alga showed that one is involved in the regulation of nitrate assimilatory genes and proteins^72,73^ and that the other is required for acclimation to hypoxia and other conditions that impact the cellular energy status^71^.

The *Chloropicon* genome sequence provides a solid foundation for future studies on the special attributes enabling the Chloropicophyceae to play key roles in phytoplankton communities of tropical open oceanic waters. It will be important to characterize at the genome-wide level the significant genetic diversity uncovered in this class^29,30^ and search for genes/pathways linked to species or ecotype distributions.

## Methods

### Cell culture and DNA/RNA extraction

*Chloropicon primus* strain CCMP 1205 (isolated in 1965 from the Atlantic Ocean) was obtained from the National Center for Marine Algae and Microbiota (East Bootbay, ME, USA) where it is maintained as a cryopreserved, genetically pure culture after it was re-cloned in October 1987. For DNA extraction, cells were grown synchronously in L1 medium at 18 °C under 12-hour light/dark cycles and harvested after 2–3 weeks. For RNA extraction, independent cultures were grown in the same medium under synchronized and continuous light conditions; synchronized cells were harvested 2 and 8 h after the onset of the light (L2 and L8) and dark (D2 and D8) cycles, whereas cells grown in continuous light were harvested at the exponential and stationary growth phases. Total DNA and RNA were extracted using the HP Plant DNA Mini and Total RNA II kits (Omega Bio-tek, Norcross, GA, USA), respectively. RNA integrity was monitored using a Bioanalyzer (Agilent Technologies, Santa Clara, CA, USA).

### DNA and RNA sequencing

The *Chloropicon* genome was sequenced using a combination of high-throughput short and long reads generated on the Illumina (San Diego, CA, USA) and Pacific Biosciences (Menlo Park, CA, USA) platforms, respectively. Paired-end (average insert size of 522 bp) and mate-pair (average insert size of 1757 bp) libraries were prepared using the Illumina TruSeq DNA kit and the mate-pair protocol of Birol et al.^74^, and MiSeq sequencing at the Plateforme d’Analyses Génomiques of Université Laval (Québec, QC, Canada) yielded 9.6 million paired-end reads of 250 bp and 6.0 million mate-pair reads of 300 bp. For long-read sequencing, DNA was sheared by centrifugation in Covaris g-TUBEs (Woburn, MA, USA), libraries were prepared with the DNA Template Prep Kit 3.0 using the 20-kb protocol (Pacific Biosciences), and DNA was sequenced on the PacBio RS II platform (four SMRT cells, P6-C4 chemistry) at the University of Michigan DNA Sequencing Core (Ann Arbor, MI), providing 1.3 GB of reads, with polymerase and subread N50s of 7.99 and 4.89 kb, respectively (Supplementary Fig. 9).

A normalized RNAseq library was prepared by the Plateforme d’Analyses Génomiques of Université Laval from a mixture containing equal amounts of RNAs isolated from the L2, L8, D2, and D8 synchronized cells and exponential and stationary cultures grown in continuous light. The library was constructed using the Illumina TruSeq RNA kit and the normalization protocol of Zhulidov et al.^75^ and sequenced on the Illumina HiSEQ 2000 platform (300 cycles) at the McGill University and Génome Québec Innovation Centre (Montréal, Canada), yielding 22,223,345 paired-end reads.

### Genome assembly

Independent de novo assemblies were generated from short and long Illumina and PacBio read datasets and then merged to produce a high-quality consensus draft using overlap-consensus-approaches followed by manual curation/inspection of discrepancies. Low-quality bases in the Illumina reads were removed with PRINSEQ 0.20.4 and adapter sequences were discarded using FASTX_CLIPPER from the FASTX-Toolkit 0.0.14 package. Pre-assembly of overlapping paired-end reads was performed with FLASH. Assembly was performed using Ray 2.3.1 and a kmer value of 61 on the paired-end and mate-pair datasets, with reads overlapped with FLASH used as singletons. PacBio assemblies were performed using MHAP 1.5 and HGAP3 as implemented in the Celera assembler version 8.3RC1 and SMRT-Analysis Portal 2.3.0, respectively. For the Celera assembly, FASTQ reads were generated from the bax.h5 files with DEXTRACTOR, converted to Celera format with the fastqToCA tool from the Celera package, quality-trimmed by overlap using runCA, and then assembled with MHAP 1.5.

Illumina Ray and PacBio HGAP3 assemblies were merged using the alignment tool for large DNA fragments implemented in Sequencher 5.4.1 (Gene Codes Corporation, Ann Arbor, MI, USA). Discrepancies in the consensus sequence were resolved manually using the editing tool of Sequencher and the basecalling of the Illumina contigs, and when indels were encountered, sequences of the long alleles were integrated into the consensus. Additional overlaps between the polished Ray/HGAP3 contigs and the Celera MHAP assembly were identified using dot plot analyses as implemented in dottup from the EMBOSS 6.4.0 package. Because the Celera assembly produced longer contigs albeit with more inaccurate basecalling, the latter contigs were only used for scaffolding the Ray/HGAP3 assemblies, with the corresponding basecalling in the scaffolded regions curated manually.

The absence of contaminants in the assembled contigs was confirmed by taxonomized BLAST homology searches against the NCBI nr/taxonomy databases using runTaxonomizedBLAST.pl.

### Genome annotations

Ribosomal and transfer RNA genes were identified using RNAmmer 1.2 and tRNAscan-SE 1.3.1, respectively. Protein-coding genes were predicted using the MAKER 2.32 and BRAKER 1.8 pipelines. For BRAKER 1.8, alignments of Illumina RNAseq reads against the assembled genome were generated in BAM format using HISAT2 2.0.1 and default parameters. To detect protein-coding genes improperly predicted or missed in previous analyses, RNAseq data were mapped independently on the assembled genome with PASS 2.23 using the default mapping seed (111111101111111) with a seed step of 3, the homopolymer flag turned on (-flc 1), an identity of 90%, a filtering length of 50 nt, and the gap mode set to 2. In addition, protein datasets derived from *Auxenochlorella protothecoides*^76^, *Bathycoccus prasinos*^5^, *Chlorella variabilis*^13^, *C. reinhardtii*^9^, *Coccomyxa subellipsoidea*^12^, *Gonium pectorale*^8^, *Helicosporidium* sp.^77^, *Klebsormidium flaccidum*^15^, *Micromonas pusilla*^6^, *Micromonas commoda*^6^, *Ostreococcus tauri*^4^, *Ostreococcus lucimarinus*^7^, and *Volvox carteri*^10^ were queried against the *Chloropicon* chromosomes using TBLASTN searches. Results from gene predictions, RNA mapping and homology searches were loaded in a local Web-Apollo 2.0.2-RC3 server using built-in and custom Perl scripts, and the annotations curated using the sum of all information and Web-Apollo built-in tools.

Curated GFF3 annotations were exported using Web-Apollo built-in tools, converted to EMBL format with WebApolloGFF3toEMBL.pl and further curated manually using Artemis 16.0.0, wherein locus tags were automatically added using the feature Edit > Automatically Create Gene Names. Amino acid sequences from predicted proteins were exported with EMBLtoPROT.pl and putative functions were inferred independently using InterProScan 5 and BLASTP homology searches against the UniProt Swiss-Prot/TrEMBL databases and the *Chlamydomonas* and *Coccomyxa* protein datasets. Curated EMBL annotations were converted to TBL format using the curated product list and EMBLtoTBL.pl. Final accessions were generated and validated with TBL2ASN.

### Gene expression analysis

RNAseq Illumina reads were mapped against the *Chloropicon* genome with PASS 2.23 using the parameters described in the previous section. The resulting alignments in SAM format were converted to BAM format, then sorted using Samtools 1.3.1. RNAseq sequencing depths for individual nucleotide positions were inferred with the depth -aa command line option of Samtools. Gene expression levels were inferred by averaging the sequencing depths of all nucleotides from the exons of the corresponding genes in the *Chloropicon* genome annotation with genes_expressed.pl.

### BUSCO analyses

BUSCO 3 was used to compare the quality and completeness of the DNA assemblies and gene annotations of *Chloropicon* with those of 12 other green algae. The annotated proteins of these algae were queried against the OrthoDB v10 database, which contains the 2168 near-universal single-copy orthologs found in the Chlorophyta.

### Variant identification and GC-content analyses

Variants were identified by mapping genomic reads onto the chromosome assemblies that were masked for repeated sequences using RepeatModeler 1.0.11 and RepeatMasker 4.0.7 with the -no_IS and -nolow options. The Illumina paired-end reads were aligned using minimap2 using the -x sr preset and both SNPs and small indels were called with VarScan2 2.4.3 using a minimum variant frequency (–min-var-freq) of 0.2 and the minimum number of supporting reads (–min-reads2) set to 50, as implemented in get_SNP.pl 1.9f from the SSRG pipeline (https://github.com/PombertLab). For all SNP positions, the nucleotides corresponding to the observed alleles together with their frequencies were extracted from the varscan2 VCF files with sort_SNPs.pl, and plots showing the distribution of SNP allelic frequencies on one or more chromosomes were generated with Rstudio 1.1.453 and R 3.5.0 using the fitdistrplus 1.0–9 package. Large indels were identified with the PacBio structural variant calling and analysis tools (PBSV 2.1.1). Chromosomal GC contents were plotted with Circos using 1000-nt window and 500-nt slide parameters with GC_content_to_Circos.pl.

### Determination of sequencing depth

Illumina reads (paired-ends, mate pairs, paired-ends + mate pairs) and PacBio subreads were aligned independently on the *Chloropicon* unmasked nuclear genome using minimap2. Sequencing depth of each chromosome was determined from the BAM alignment files with the Samtools depth function as implemented in get_SNPs.pl. The same methodology was used to calculate the PacBio sequencing depth of the chloroplast and mitochondrial genomes (GenBank accession KJ746601 and MK085998, respectively). Sequencing depths were plotted with Circos using 10,000-nt window and 5000-nt slide parameters with Coverage_to_Circos.pl.

### Comparative analyses of gene content and gene order

Putative orthologs of the *Chloropicon* gene products in the 13 green algae mentioned in the section Genome annotations were searched by homology searches using BLASTP (*E*-value cutoff = 1.0E-10) and TBLASTN (*E*-value cutoff = 1.0E-10). BLASTP and TBLASTN hits were parsed at the desired cutoff using shared_proteins.pl. Maps of metabolic pathway were retrieved from the KEGG online repository^78^ with KEGG.sh, and KEGG orthologs (KOs) were assigned for each algal dataset with GhostKOALA. The identified KOs were concatenated into distinct KEGG pathway matrices with KOs_to_matrices.pl and plotted using the Bioconductor ComplexHeatmap package as implemented in R 3.5.0 with MatrixR_plotter.pl.

Gene order data were extracted from the GFF annotation files of the compared algal genomes with gff_to_synteny.pl using regular expressions tailored to account for the variations between GFF files. Orthologous proteins encoded by shared gene pairs were identified by BLASTP searches with get_synteny.pl, iteratively allowing for 0, 1, 5, 10, and 50 genes interspersed between potential pairs. In this analysis, the genes in each potential shared pair had to be in the same relative orientation in the compared genomes, present on a single contig/chromosome in each genome (highly fragmented genomes will inherently produce fewer gene pairs with this assumption), and distant by no more than the number of genes allowed in-between (genes were allowed in-between to account for indels and for inaccurate/spurious predictions resulting in low homology or extraneously predicted products). Gene colocalization on similar chromosomes or contigs between genomes was investigated with chromosome_explorer.pl and the output was converted to matrices with get_matrix.pl. Gene colocalization data were plotted using the Bioconductor ComplexHeatmap package as implemented in R 3.4.0 with script contatenate_matrices.pl.

### Phylogenetic analyses

To determine the phylogenetic relationships of *Chloropicon* with the 12 algae selected for comparative genome analyses, a maximum likelihood tree was inferred from the proteins encoded by the orthologous single-copy genes present in all species. These genes were identified using Orthofinder 2.3.1; individual protein products were aligned with MAFFT 7.407 using the L-INS-i method; unambiguously aligned regions of each alignment were removed using BMGE 1.12 and default parameters; and finally a supermatrix was created with create_supermatrix.pl. The resulting dataset was analyzed using IQ-TREE 1.6.7 and the GTR20 + R4 model. Separate phylogenies were also inferred from the *Chloropicon* PrpB, PrpC, PrpD and PrpF proteins and their orthologs from the NCBI nr database. Protein alignments were carried out with MAFFT 7.313 and the–auto option, and after filtration of each alignment using TrimAl 1.3 and the block = 6, gt = 0.7, st = 0.005 and sw = 3 options, trees were inferred with IQ-TREE 1.6.1 using the automatically selected best-fit model. For all trees, confidence of branch points was estimated by ultrafast bootstrap analysis with 1000 replicates^79^.

### Reporting summary

Further information on research design is available in the Nature Research Reporting Summary linked to this article.

## Supplementary information

Supplementary Information

Reporting Summary

Description of Additional Supplementary Files

Supplementary Data 1

Supplementary Data 2

Supplementary Data 3

Supplementary Data 4

Supplementary Data 5

Supplementary Data 6

Supplementary Data 7

Supplementary Data 8

Supplementary Data 9

Supplementary Data 10

Supplementary Data 11

Supplementary Data 12

Supplementary Data 13

Supplementary Data 14

Supplementary Data 15

Supplementary Data 16

Source Data file

## Data Availability

Data supporting the findings of this work are available within the paper and its Supplementary Information files. A reporting summary for this Article is available as a Supplementary Information file. The datasets generated and analyzed during the current study are available from the corresponding author upon request. The annotated *Chloropicon* genome sequence has been deposited in the NCBI Genbank database (accessions CP031034-CP031053). All raw DNA sequencing (SRR8185492-SRR8185497) and RNAseq (SRR8992761) data have been submitted to the NCBI Sequence Read Archive. All these data are accessible under the NCBI Bioproject PRJNA316521 [https://www.ncbi.nlm.nih.gov/bioproject/PRJNA316521]. The source data underlying Figs. 1d, 4a–d and 5a–c are provided as a Source Data file.

## Code availability

All custom Perl scripts used in this study are available on the Pombert Lab GitHub page [https://github.com/PombertLab].
