# Supplementary material for: A streamlined and predominantly diploid genome in the tiny marine green alga Chloropicon primus
Source: Nat Commun. 2019 Sep 6;10:4061. doi: 10.1038/s41467-019-12014-x (PMC6731263; doi:10.1038/s41467-019-12014-x)
Supplement: Supplementary file 1 — Supplementary Information [file 41467_2019_12014_MOESM1_ESM.pdf]

A streamlined and predominantly diploid genome in the tiny marine green alga *Chloropicon primus*

Lemieux *et al.*

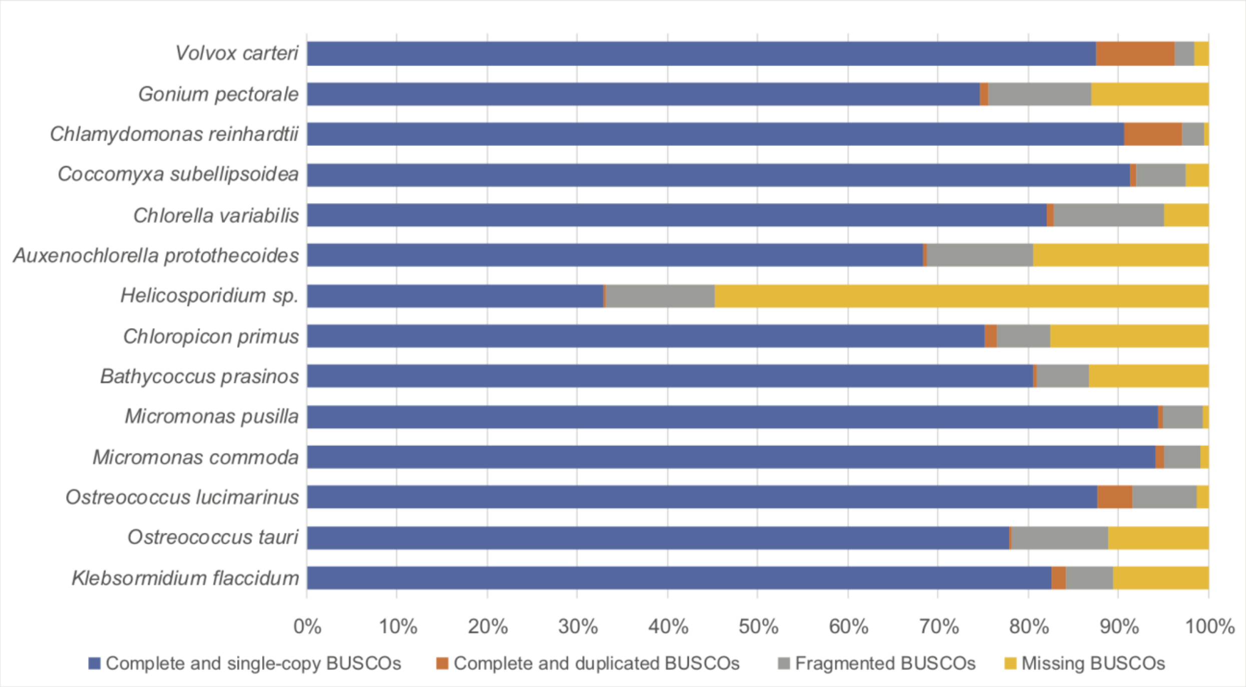

Supplementary Figure 1. BUSCO assessment of sequence assembly completeness of the *Chloropicon* and 13 other green algal genomes.

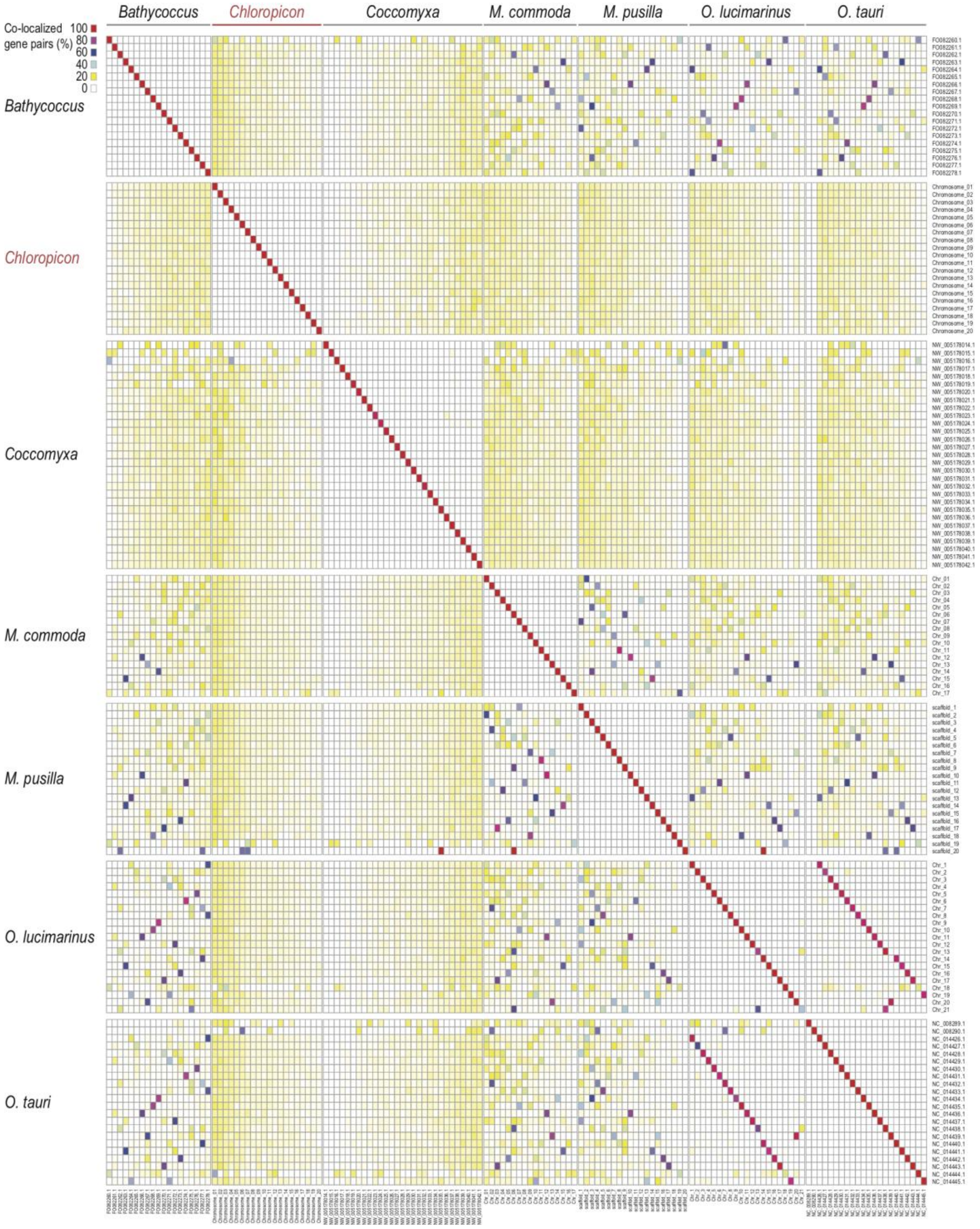

Supplementary Figure 2. Comparative analysis of gene order in *Chloropicon* and other green algal genomes. Only complete genomes or genomes displaying little fragmentation, i.e. a few contigs or scaffolds, were selected here. For each chromosome or DNA assembly of a given alga (rows), the proportions of protein-coding gene pairs sharing the same order and polarity relative to the individual chromosomes or DNA assemblies of the compared algae (columns) were color-coded accordingly.

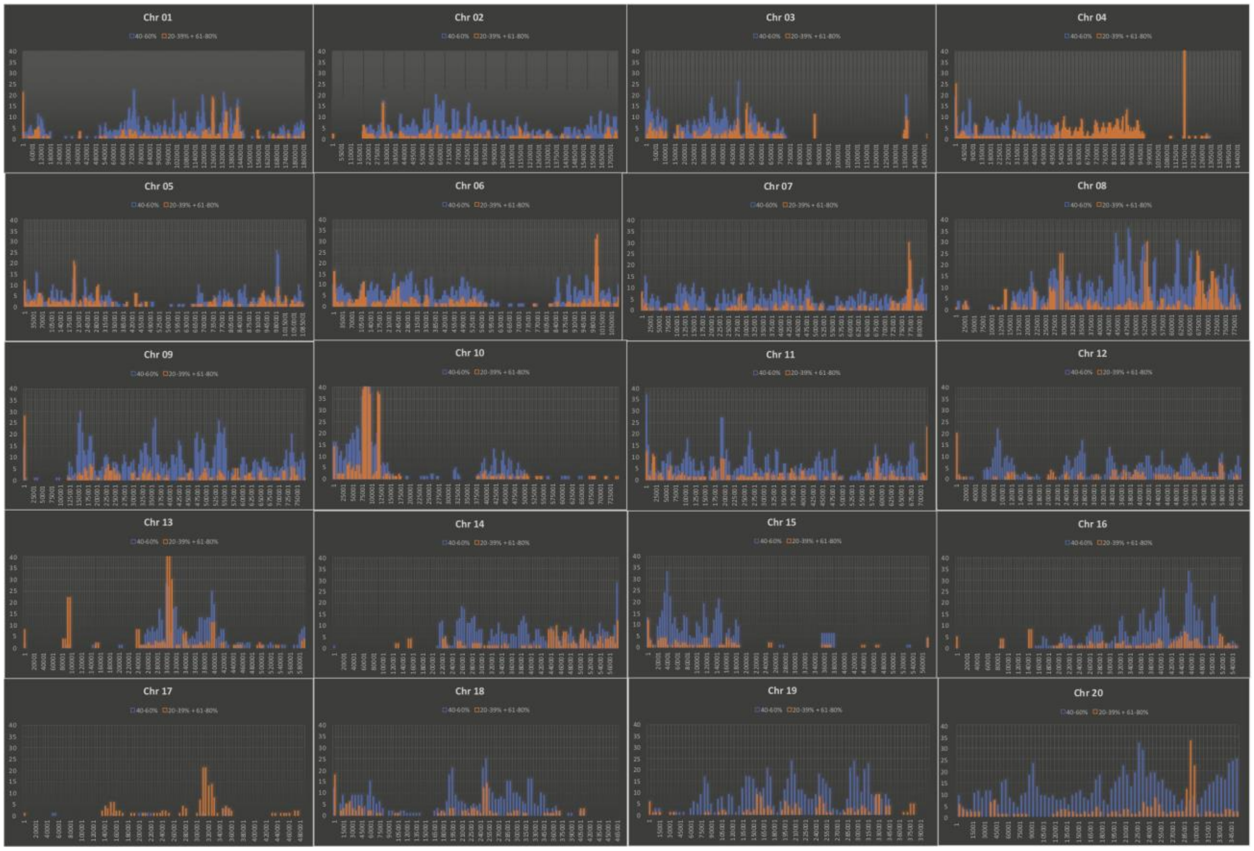

Supplementary Figure 3. Chromosomal locations of polymorphisms belonging to two categories of allelic frequencies. Polymorphisms types include SNP and small indels. 40-60%: blue; 20-39% + 61-80%: orange.

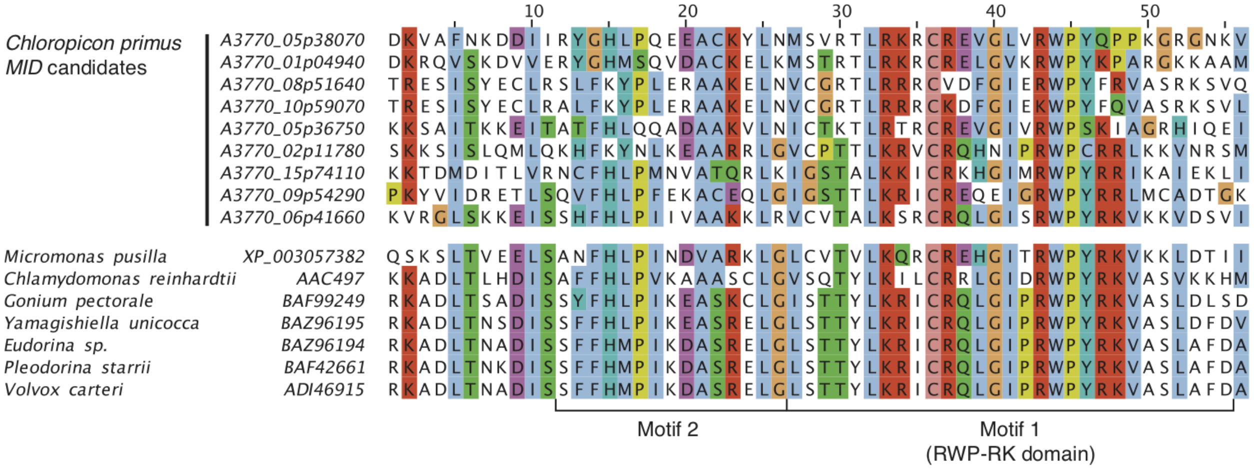

Supplementary Figure 4. Sequence conservation of motifs 1 and 2 among the nine putative RWP-RK transcription factors identified in *Chloropicon* and the *Mid* proteins of *Micromonas* and volvocine algae.

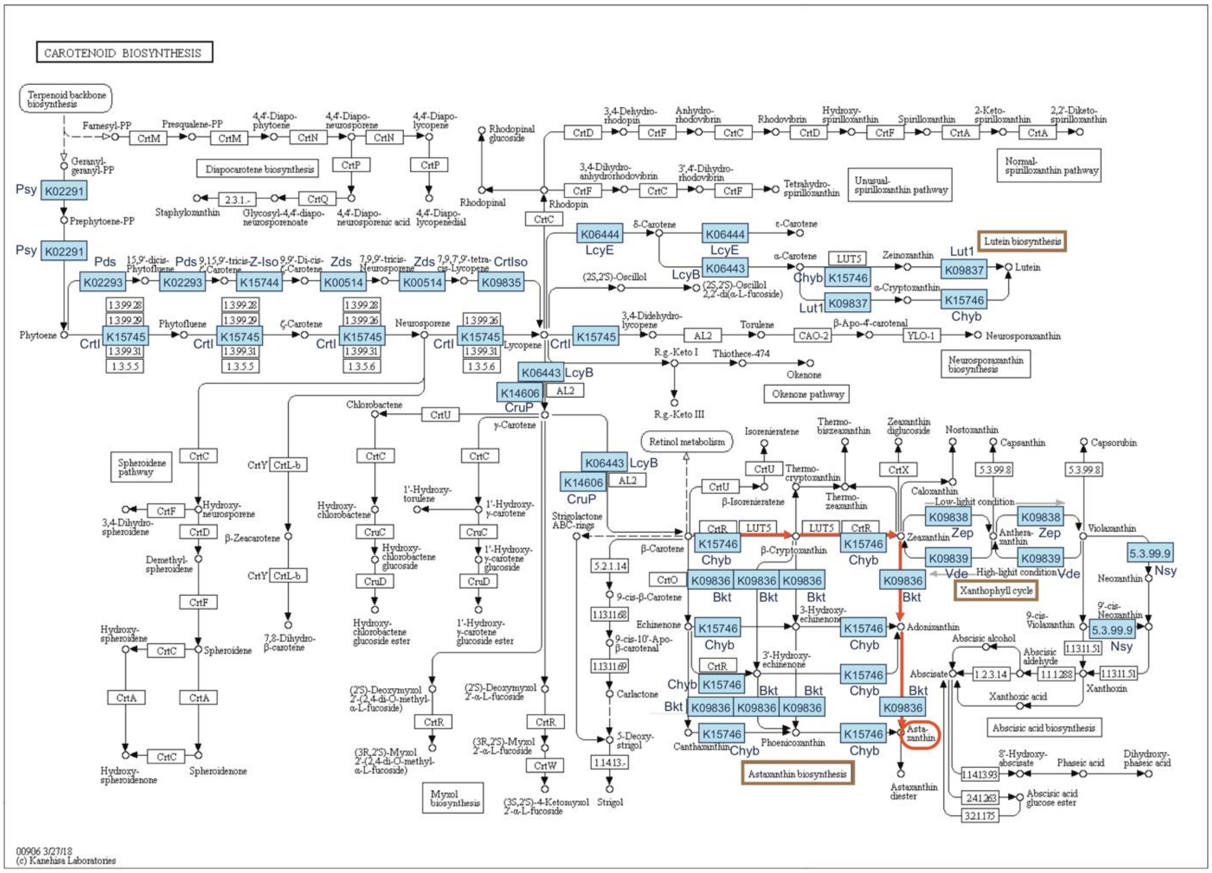

Supplementary Figure 5. Mapping of *Chloropicon* predicted proteins on the KEGG pathways for carotenoid biosynthesis (ko00906). Each protein is represented as a blue box, with the assigned KO indicated within the box. Supplementary Data 12 reports the positions of the encoding genes, protein names, presence/absence of homologs in compared green algae as well as other information, including expression data and predicted cellular localization.

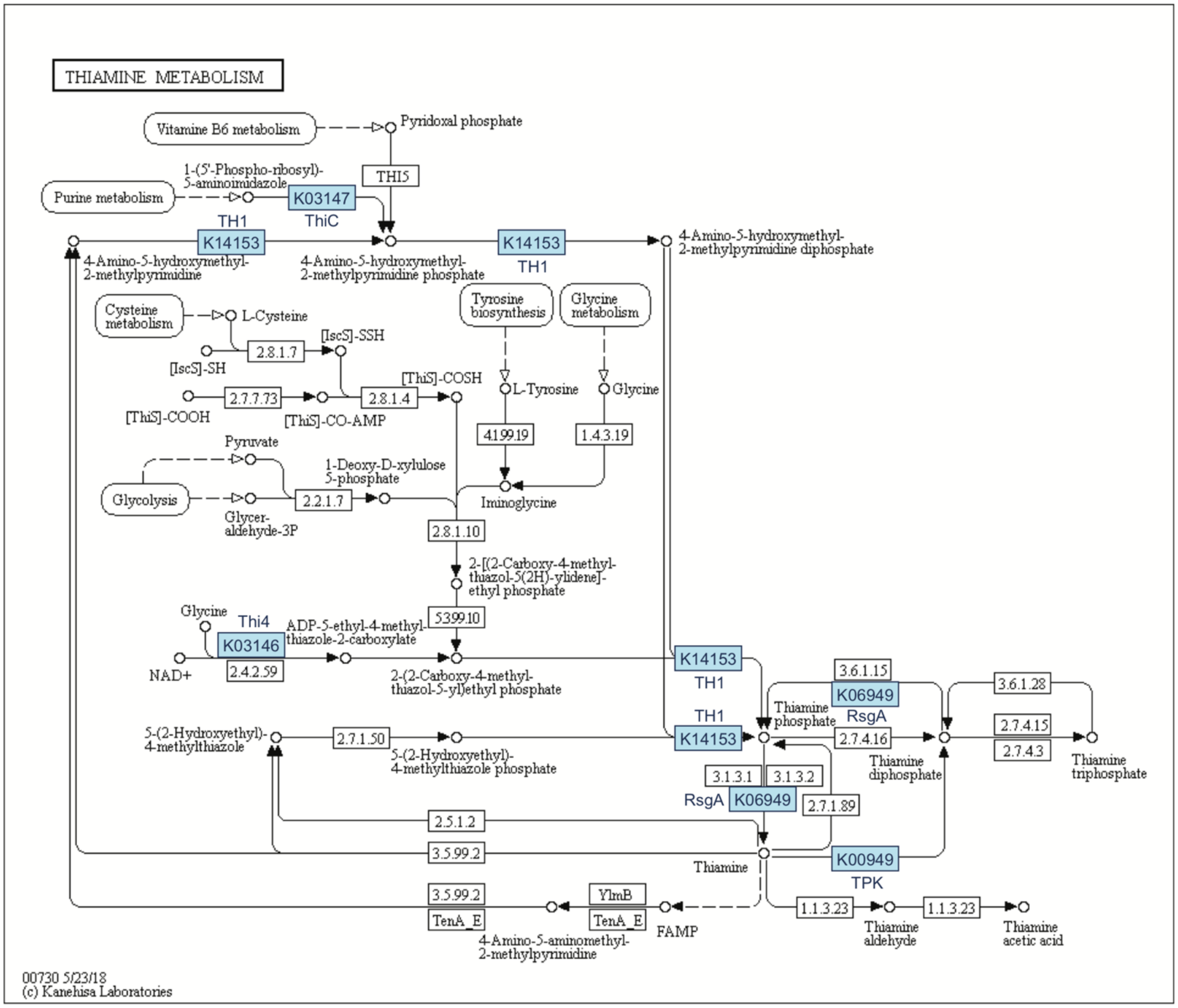

Supplementary Figure 6. Mapping of *Chloropicon* predicted proteins (blue boxes) on the KEGG pathways for thiamine biosynthesis (ko00730). Supplementary Data 13 reports the positions of the encoding genes, protein names, presence/absence of homologs in compared green algae as well as other information, including expression data and predicted cellular localization.

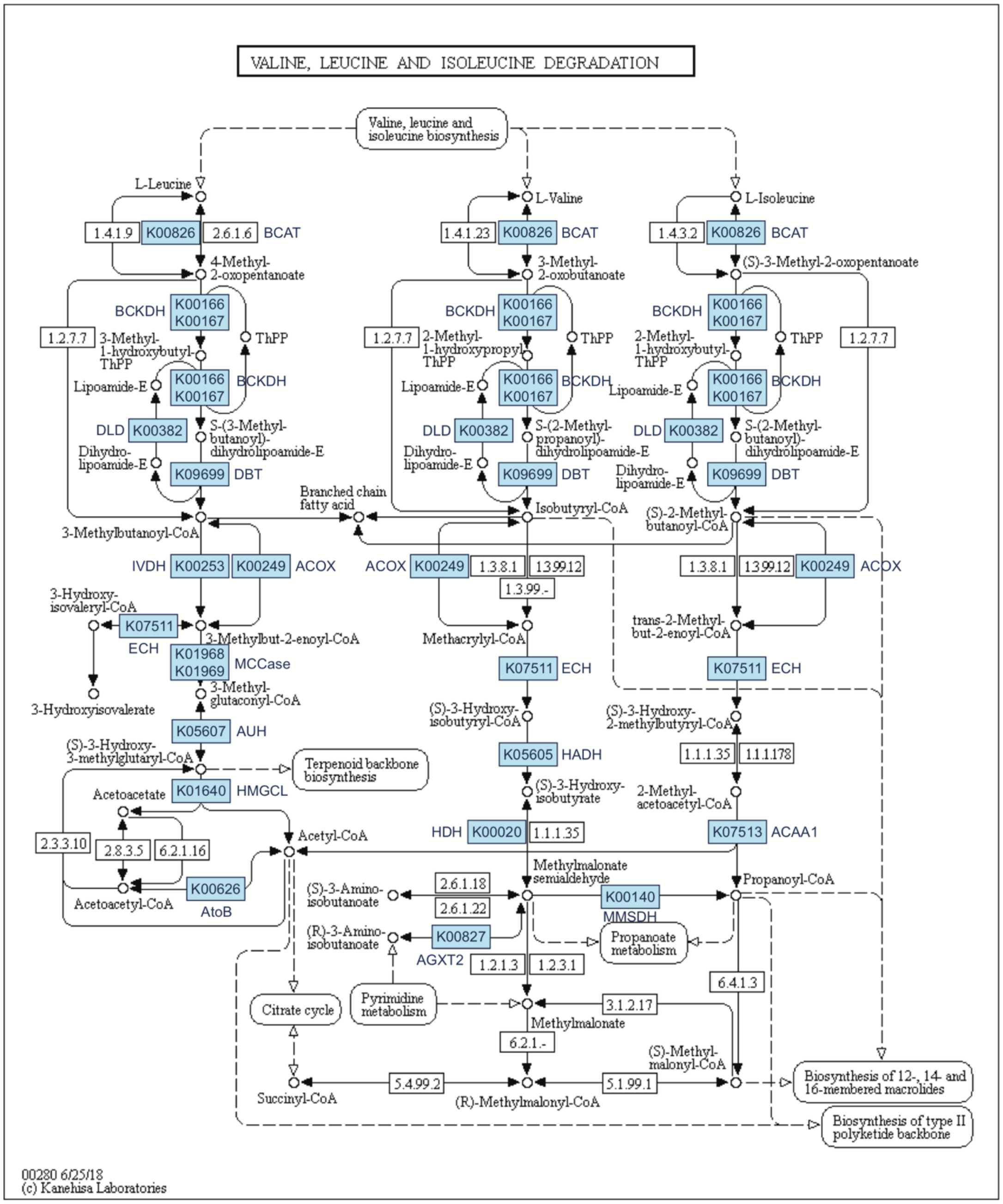

Supplementary Figure 7. Mapping of *Chloropicon* predicted proteins (blue boxes) on the KEGG pathways for BCAA catabolism (ko00280). Note that presence of the enzyme catalysing the conversion of 3-hydroxy-2-methylbutyryl-CoA to 2-methyl-acetoacetyl-CoA [1.1.1.35/1.1.1.178] in the isoleucine pathway could not be inferred because no protein with this activity has been experimentally validated and annotated in any member of the Chloroplastida. Supplementary Data 14 reports the positions of the encoding genes, protein names, presence/absence of homologs in compared green algae as well as other information, including expression data and predicted cellular localization.

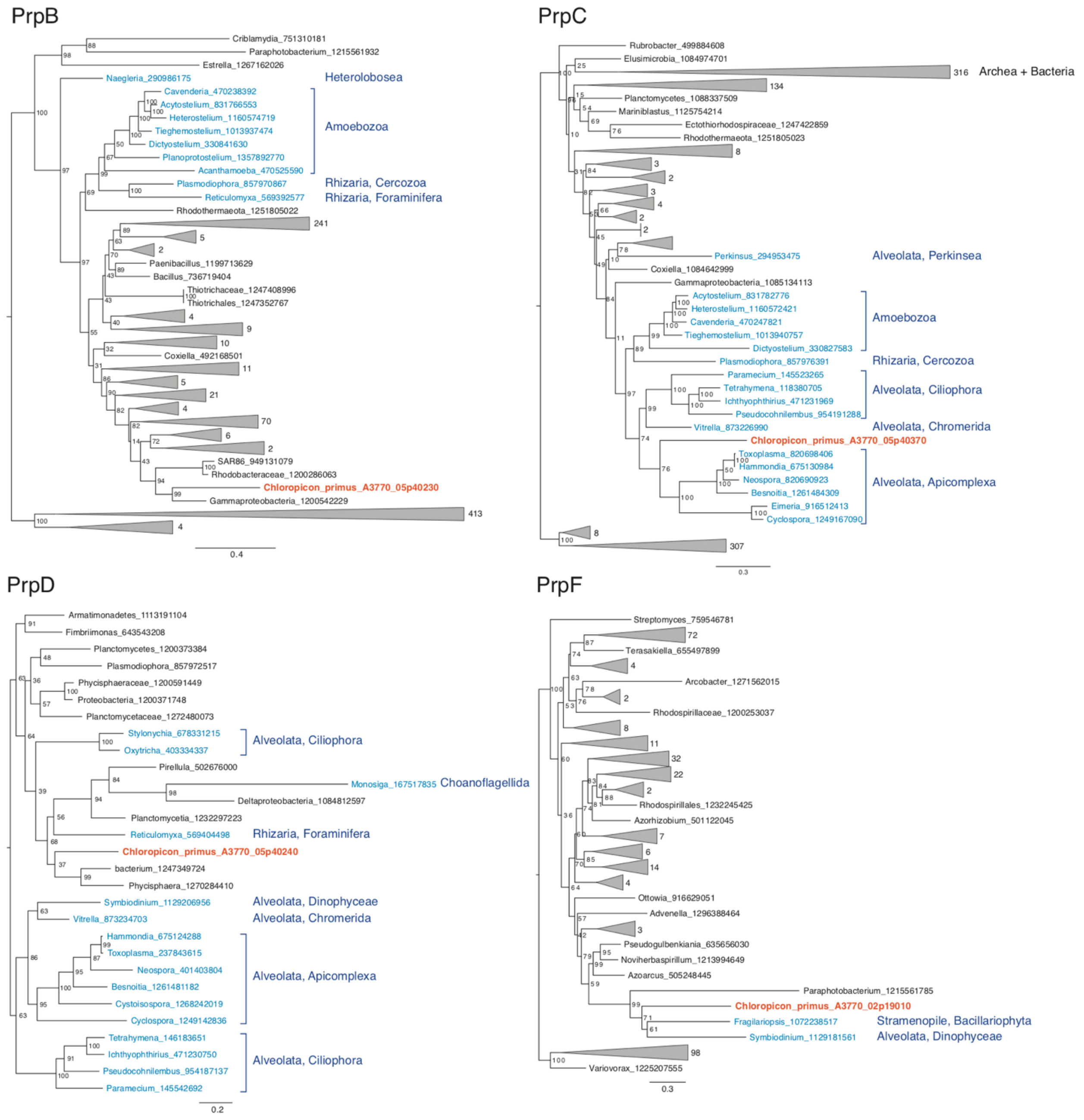

Supplementary Figure 8. Phylogenetic relationships of the *Chloropicon* PrpB, PrpC, PrpD and PrpF proteins with their prokaryotic and eukaryotic homologs. To infer these relationships, maximum likelihood trees were constructed using IQ-TREE; bootstrap support values are reported on the nodes of the consensus trees. Prokaryotic clades with more than 90% bootstrap support were collapsed (grey triangles) to simplify the topologies; the numbers of taxa in these clades are indicated on the right of the triangles. Eukaryotic taxa are shown in blue.

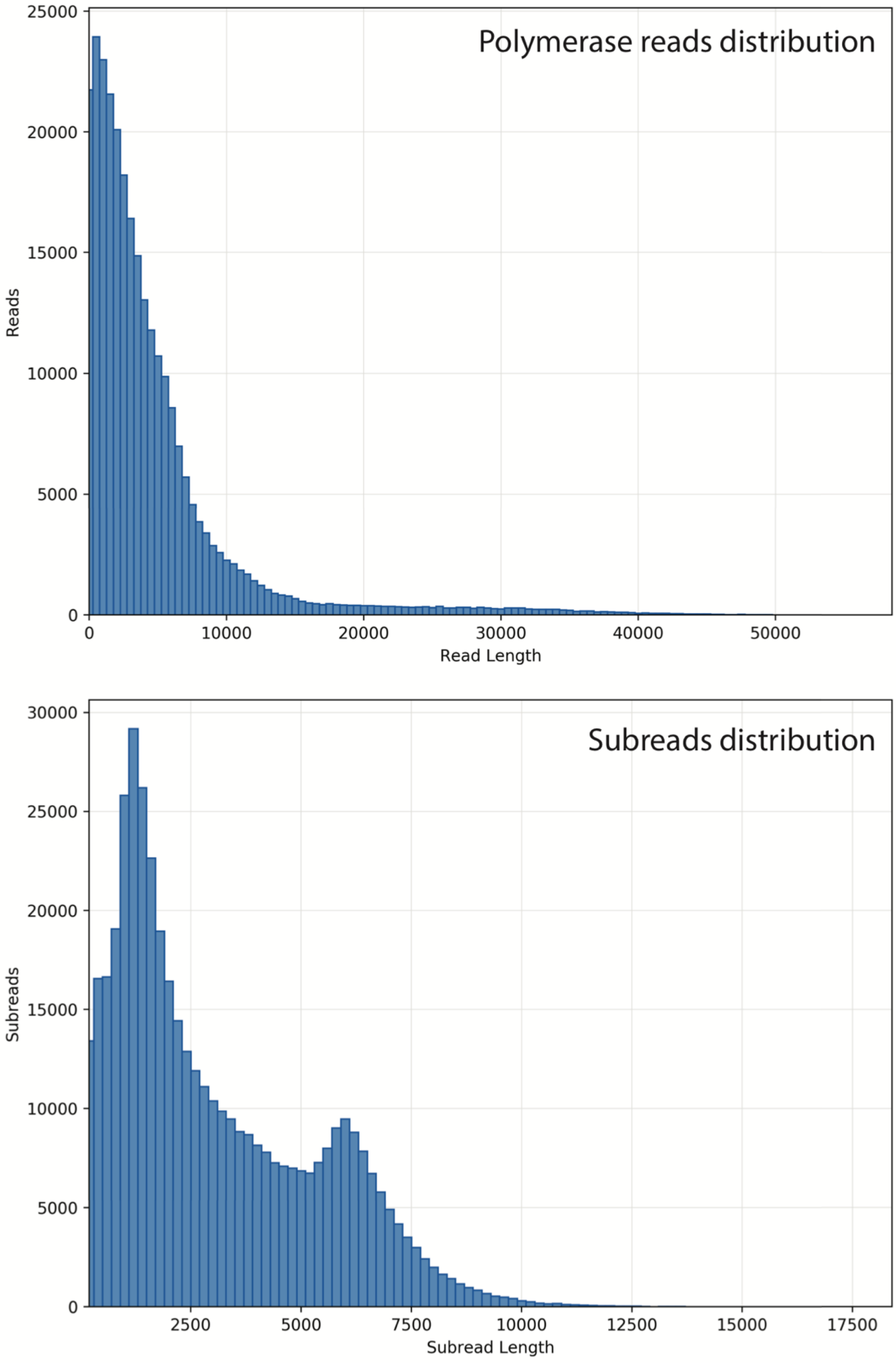

Supplementary Figure 9. PacBio polymerase read and subread length distributions.
