# Supplementary material for: A streamlined and predominantly diploid genome in the tiny marine green alga Chloropicon primus
Source: Nat Commun. 2019 Sep 6;10:4061. doi: 10.1038/s41467-019-12014-x (PMC6731263; doi:10.1038/s41467-019-12014-x)
Supplement: Supplementary file 2 — Reporting Summary [file 41467_2019_12014_MOESM2_ESM.pdf]

nature research

Corresponding author(s): Claude LEMIEUX

Last updated by author(s): Aug 13, 2019

Reporting Summary

Nature Research wishes to improve the reproducibility of the work that we publish. This form provides structure for consistency and transparency in reporting. For further information on Nature Research policies, see Authors & Referees and the Editorial Policy Checklist .

Statistics

For all statistical analyses, confirm that the following items are present in the figure legend, table legend, main text, or Methods section.

n/a	Confirmed	
☒	☐	The exact sample size (*n*) for each experimental group/condition, given as a discrete number and unit of measurement
☒	☐	A statement on whether measurements were taken from distinct samples or whether the same sample was measured repeatedly
☒	☐	The statistical test(s) used AND whether they are one- or two-sided<br>*Only common tests should be described solely by name; describe more complex techniques in the Methods section.*
☒	☐	A description of all covariates tested
☒	☐	A description of any assumptions or corrections, such as tests of normality and adjustment for multiple comparisons
☒	☐	A full description of the statistical parameters including central tendency (e.g. means) or other basic estimates (e.g. regression coefficient) AND variation (e.g. standard deviation) or associated estimates of uncertainty (e.g. confidence intervals)
☒	☐	For null hypothesis testing, the test statistic (e.g. *F*, *t*, *r*) with confidence intervals, effect sizes, degrees of freedom and *P* value noted<br>*Give P values as exact values whenever suitable.*
☒	☐	For Bayesian analysis, information on the choice of priors and Markov chain Monte Carlo settings
☒	☐	For hierarchical and complex designs, identification of the appropriate level for tests and full reporting of outcomes
☒	☐	Estimates of effect sizes (e.g. Cohen's *d*, Pearson's *r*), indicating how they were calculated

Our web collection on statistics for biologists contains articles on many of the points above.

Software and code

Policy information about availability of computer code

Data collection	No software was used to collect data in this study.
Data analysis	The only commercial software used for data analysis in this study is Sequencher 5.4.1 (Gene Codes Corporation, Ann Arbor, MI, USA).<br><br>All other software tools employed are open source; they include Artemis 16.0.0, BLASTP 2.2.31+, Bowtie2 2.3.1, BRAKER 1.8, BUSCO 3, Circos 0.69.6, Dextractor 1.0p1, dottup in EMBOSS 6.4.0, FASTX-Toolkit 0.0.14, fitdistrplus 1.0-9, FLASH 1.2.11, GhostKOALA 2.0, HGAP3 in the SMRT-Analysis Portal 2.3.0, HISAT2 2.0.1, InterProScan 5, IQ-TREE 1.6.1, MAFFT 7.313, MAKER 2.32, MHAP 1.5, runCA and fastqToCA in the Celera assembler version 8.3RC1, Minimap2, Orthofinder 2.3.1, PASS 2.23, PBSV 2.1.1, PRINSEQ 0.20.4, R 3.5.0, Ray 2.3.1, RepeatMasker 4.0.7, RepeatModeler 1.0.11, RNAmmer 1.2, Rstudio 1.1.453, Samtools 1.3.1, TBL2ASN 25.6, TBLASTN 2.2.31+, TrimAl 1.3, tRNAscan-SE 1.3.1, VarScan2 2.4.3, and Web-Apollo 2.0.2-RC3.<br><br>All custom Perl scripts used in this study are freely available on the GitHub page of the Pombert Lab (https://github.com/PombertLab), in the Publication_scripts/2019_Nature_Communications_Chloropicon_primus/ directory. The full names of the scripts essential to this study are as follows: chromosome_explorer.pl, contatenate_matrices.pl, EMBLtoPROT.pl, EMBLtoTBL.pl, GC_content_to_Circos.pl, genes_expressed.pl, get_matrix.pl, get_SNP.pl, get_synteny.pl, gff_to_synteny.pl, KOs_to_matrices.pl, MatrixR_plotter.pl, Meth_to_Circos.pl, runTaxonomizedBLAST.pl, shared_proteins.pl, sort_SNPs.pl, VCF_to_Circos.pl, WebApolloGFF3toEMBL.pl. All miscellaneous scripts used to parse and/or reformat datasets but otherwise not essential to the analyses are also included in this directory. The raw output from GHOSTKOALA analyses (CCMP_user_ko_definition.txt) and the annotated list of Chloropicon primus products (Verified_products_ALL.list) are also available in this directory.

For manuscripts utilizing custom algorithms or software that are central to the research but not yet described in published literature, software must be made available to editors/reviewers. We strongly encourage code deposition in a community repository (e.g. GitHub). See the Nature Research guidelines for submitting code & software for further information.

Data

Policy information about availability of data

All manuscripts must include a data availability statement. This statement should provide the following information, where applicable:

- Accession codes, unique identifiers, or web links for publicly available datasets
- A list of figures that have associated raw data
- A description of any restrictions on data availability

The annotated Chloropicon genome sequence has been deposited in the NCBI Genbank database (accessions CP031034-CP031053) and all raw DNA sequencing (SRR8185492-SRR8185497) and RNAseq (SRR8992761) data have been submitted to the NCBI Sequence Read Archive. All these data are accessible under the NCBI Bioproject PRJNA316521 [https://www.ncbi.nlm.nih.gov/bioproject/PRJNA316521].

Supplementary Data 2 provides the list of the predicted protein-coding genes in the Chloropicon genome and the following information: genomic locus, gene product predicted by GhostKOALA and BLASTP searches, expression data, and presence/absence of homologs in other green algae (E-value cutoff = 1.0E-10) along with recorded E-values. Algal datasets used in this study and described in Supplementary Data 1 were retrieved from NBCI, Phytozome v11 (https://phytozome.jgi.doe.gov/pz/portal.html) and ORCAE (http://bioinformatics.psb.ugent.be/orcae/). Supplementary Data 7 and 8 provide the localizations and allelic frequencies of SNPs and indels in the Chloropicon genome.

Field-specific reporting

Please select the one below that is the best fit for your research. If you are not sure, read the appropriate sections before making your selection.

☒ Life sciences ☐ Behavioural & social sciences ☐ Ecological, evolutionary & environmental sciences

For a reference copy of the document with all sections, see nature.com/documents/nr-reporting-summary-flat.pdf

Life sciences study design

All studies must disclose on these points even when the disclosure is negative.

Sample size	Chloropicon primus is a singular organism. Its genome was sequenced from the only axenic culture currently available for this alga (strain CCMP 1205 from the National Center for Marine Algae and Microbiota, East Bootbay, ME, USA). RNA was extracted at different time points from independently grown synchronized cultures.
Data exclusions	No data were excluded in this study.
Replication	All attempts to replicate the analyses described in this study were successful.
Randomization	Randomization is not relevant to our study because only one organism was analyzed.
Blinding	Blinding is not relevant to our study because only one organism was analyzed.

Reporting for specific materials, systems and methods

We require information from authors about some types of materials, experimental systems and methods used in many studies. Here, indicate whether each material, system or method listed is relevant to your study. If you are not sure if a list item applies to your research, read the appropriate section before selecting a response.

Materials & experimental systems

n/a	Involved in the study	
☒	☐	Antibodies
☒	☐	Eukaryotic cell lines
☒	☐	Palaeontology
☒	☐	Animals and other organisms
☒	☐	Human research participants
☒	☐	Clinical data

Methods

n/a	Involved in the study	
☒	☐	ChIP-seq
☒	☐	Flow cytometry
☒	☐	MRI-based neuroimaging
