# Supplementary material for: A streamlined and predominantly diploid genome in the tiny marine green alga Chloropicon primus
Source: Nat Commun. 2019 Sep 6;10:4061. doi: 10.1038/s41467-019-12014-x (PMC6731263; doi:10.1038/s41467-019-12014-x)
Supplement: Supplementary file 3 — Description of Additional Supplementary Files [file 41467_2019_12014_MOESM3_ESM.docx]

**Description of Additional Supplementary Files**

File Name: Supplementary Data 1.

Description: Genome statistics for *Chloropicon* and the 12 other green algae selected for comparative analyses.

File Name: Supplementary Data 2.
Description: List of predicted protein-coding genes in the *Chloropicon* genome.

File Name: Supplementary Data 3.
Description: Polyketide synthase genes in the *Chloropicon* genome.

File Name: Supplementary Data 4.
Description: Sialyltransferase and sialidase genes in the *Chloropicon* genome.

File Name: Supplementary Data 5.

Description: Guanylyl cyclase genes in the *Chloropicon* genome.

File Name: Supplementary Data 6.

Description: Putative transposase genes in the *Chloropicon* genome.

File Name: Supplementary Data 7.

Description: Positions and allelic frequencies of SNPs and small indels (≤ 43 bp) in the *Chloropicon* genome.

File Name: Supplementary Data 8.

Description: Localisations and allelic frequencies of indels ≥ 20 bp in the *Chloropicon* genome.

File Name: Supplementary Data 9.

Description: Meiotic recombination genes in the *Chloropicon* genome. File Name: Supplementary Data 10.

Description: Flagella-related genes in the *Chloropicon* genome.

File Name: Supplementary Data 11.

Description: Lhca and Lhcb genes in the *Chloropicon* genome.

File Name: Supplementary Data 12.

Description: Carotenoid biosynthesis genes in the *Chloropicon* genome.

File Name: Supplementary Data 13.

Description: Thiamine biosynthesis genes in the *Chloropicon* genome.

File Name: Supplementary Data 14.

Description: BCAA catabolic genes in the *Chloropicon* genome.

File Name: Supplementary Data 15.

Description: Propionate catabolic genes in the *Chloropicon* genome.

File Name: Supplementary Data 16.

Description: Genes involved in anaerobic fermentation in the *Chloropicon* genome.
